# Genomic instability induced by radiation-mimicking chemicals is not associated with persistent mitochondrial degeneration

**DOI:** 10.1007/s00411-021-00927-5

**Published:** 2021-07-30

**Authors:** Jukka Luukkonen, Anne Höytö, Miiko Sokka, Juhani Syväoja, Jukka Juutilainen, Jonne Naarala

**Affiliations:** 1 Department of Environmental and Biological Sciences, University of Eastern Finland, Yliopistonranta 1, P.O. Box 1627, 70211 Kuopio, Finland; 2 STUK-Radiation and Nuclear Safety Authority, Helsinki, Finland; 3 Department of Environmental and Biological Sciences, University of Eastern Finland, Joensuu, Finland; 4 Department of Molecular Biology, Cell Biology and Biochemistry, Brown University, Providence, RI USA; 5 Institute of Biomedicine, University of Eastern Finland, Kuopio, Finland

**Keywords:** Induced genomic instability, Reactive oxygen species, Ionizing radiation mimics, Mitochondria, Micronuclei

## Abstract

Ionizing radiation has been shown to cause induced genomic instability (IGI), which is defined as a persistently increased rate of genomic damage in the progeny of the exposed cells. In this study, IGI was investigated by exposing human SH-SY5Y neuroblastoma cells to hydroxyurea and zeocin, two chemicals mimicking different DNA-damaging effects of ionizing radiation. The aim was to explore whether IGI was associated with persistent mitochondrial dysfunction. Changes to mitochondrial function were assessed by analyzing mitochondrial superoxide production, mitochondrial membrane potential, and mitochondrial activity. The formation of micronuclei was used to determine immediate genetic damage and IGI. Measurements were performed either immediately, 8 days, or 15 days following exposure. Both hydroxyurea and zeocin increased mitochondrial superoxide production and affected mitochondrial activity immediately after exposure, and mitochondrial membrane potential was affected by zeocin, but no persistent changes in mitochondrial function were observed. IGI became manifested 15 days after exposure in hydroxyurea-exposed cells. In conclusion, immediate responses in mitochondrial function did not cause persistent dysfunction of mitochondria, and this dysfunction was not required for IGI in human neuroblastoma cells.

## Introduction

Exposure to ionizing radiation is known to result in induced genomic instability (IGI), a state of increased genomic damage evident in many cell generations after the initial insult (Morgan et al. 1996; Baverstock 2000). Genomic instability is believed to play a critical role also in carcinogenesis (Negrini et al. 2010; Burrell et al. 2013). It should be noted that genomic instability induced by environmental agents (IGI, the topic of the present paper) cannot be directly equated to genomic instability observed in cancer cells, although it is likely that the former can lead to the latter. The first IGI observations were made using ionizing radiation, but more recent studies have indicated that genomic instability can be induced by several other agents, such as chemicals, heavy metals, and non-ionizing radiation (O’Reilly and Mothersill 1997; Brennan and Schiestl 2001; Coen et al. 2001; Li et al. 2001; Phillipson et al. 2002; Cheng 2009; Korkalainen et al. 2012; Luukkonen et al. 2014). However, the dose–response of IGI is not well understood, and there is no clear understanding of cellular changes involved in the initiation of IGI or its maintenance over cell generations.

Mitochondria are central players in many cellular functions, such as apoptosis, proliferation, and energy production, so their proper functioning is crucial for cells. Importantly, mitochondrial dysfunction has been implicated in sustaining IGI (Kim et al. 2006a, b), and such a link between mitochondrial dysfunction and IGI would be understandable given the importance of mitochondria. Dysfunctional mitochondria can lead to increased levels of reactive oxygen species (ROS) (de Moura et al. 2010; Gasparre et al. 2013), which in turn could contribute to an accumulation of genomic damage. Although a connection between mitochondrial dysfunction and IGI seems reasonable, experimental evidence to assess this hypothesis is currently not adequate.

Here, we explored the possibility of IGI initiation using two chemicals that mimic the DNA-damaging effects of ionizing radiation, and investigated whether the loss of mitochondrial integrity is required for IGI. The two chemicals have different modes of action: hydroxyurea, an antineoplastic drug that inhibits DNA replication by depleting the nucleotide pool (Collins and Oates 1987; Hall et al. 2017) and zeocin, an antibiotic that causes DNA double-strand breaks (Chankova et al. 2007). As exposure of cell cultures to radiation would inevitably activate both damage mechanisms, the use of these two chemicals instead of radiation allowed us to differentiate between the effects of the two damage pathways. We used the presence of micronuclei, an easily detectable indicator of chromosomal injuries, to assess both immediate genomic damage and genomic instability persisting 8 days and 15 days following the initial exposure. Micronucleus frequency was selected as an endpoint because it has previously been used widely as a marker for IGI (Jamali and Trott 1996; Seoane et al. 2007; Yokota et al. 2010; Sciandrello et al. 2011; Korkalainen et al. 2012; Luukkonen et al. 2014). Mitochondrial dysfunction was evaluated by measuring mitochondrial superoxide (O_2_^•−^) formation, mitochondrial membrane potential, and mitochondrial activity.

## Materials and methods

### Cell culture and exposure to chemicals

**Fig. 1:**
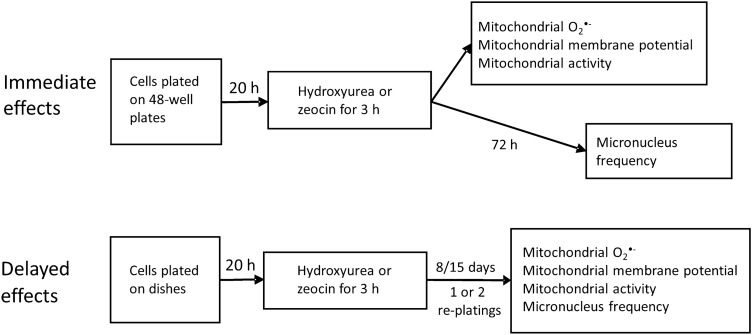
An overview of the experiments

Human SH-SY5Y neuroblastoma cells (obtained from Dr. Sven Påhlman, University of Uppsala, Sweden) were used as an experimental model, as we have previously observed IGI in this cell line (Luukkonen et al 2014). The SH-SY5Y cells were grown in Dulbecco’s Modified Eagle Medium (DMEM, 4.5 g/L glucose), supplemented with 10% inactivated FBS, 50 U/mL penicillin and 50 µg/mL streptomycin. Cell cultures were maintained in plastic bottles (75 cm^2^, Nunc, Denmark) that were kept in a humidified cell culture incubator (Heraeus HERAcell, Germany) at 37 °C with 5% CO_2_. Cells were cultured either in 48-well plates (Costar, USA) for the experiments measuring immediate effects or Petri dishes (Ø 60 mm, Nunc, Denmark) for measuring delayed effects. The number of cells used for each experiment is presented in Table 1. Cells were pre-cultivated for 20 h. An overview of the experimental protocol is presented in Fig. 1. After exposure, cells in the 48-well plates were assayed immediately, except for the cells used in the micronucleus assay, which were allowed to divide for 72 h before assaying. In the experiments for delayed time points, cells were cultured for 8 or 15 days after exposure. During the 8 and 15 days incubations for mitochondrial endpoints, cells were reseeded into new dishes once or twice, respectively. For the micronucleus assay, cells were reseeded to new dishes once during the 15 days incubation. In assays of mitochondrial endpoints, the cells were transferred to 48-well plates 24 h before the assay (0.2 × 10^6^ cells/well). For the micronucleus assays, this transfer was performed 5 days prior to the assay (0.2 × 10^6^ cells/well).

**Table 1:** The concentrations of hydroxyurea and zeocin and the number of cells seeded in the experiments

	Immediate effects		Delayed effects	
	Mitochondrial endpoints	Micronucleus assay	Mitochondrial endpoints	Micronucleus assay
Hydroxyurea, mM	0.001, 0.01, 0.05, 0.1, 0.5, 1, 5, 10	0.001, 0.01, 0.1, 1, 5, 10, 50, 100	0.1, 1, 5, 10	0.5, 5, 10, 50
Zeocin, mg/mL	0.001, 0.01, 0.05, 0.1, 0.5, 1, 5, 10	0.0001, 0.0005, 0.001, 0.005, 0.01, 0.05, 0.1	0.001, 0.01, 0.05, 0.1	0.001, 0.01, 0.05, 0.1
Cell numbers (× 10^6^)	0.20 (48-well plates)	0.15 (48-well plates)	1.8 (Petri dishes)	1.8 (Petri dishes)

Cell cultures were exposed to zeocin (Invitrogen, Carlsbad, CA, USA) or hydroxyurea (Sigma, Steinheim, Germany) for 3 h. The concentrations of the chemicals used in the experiments are presented in Table 1. The concentrations and exposure times for immediate mitochondrial endpoints were selected based on preliminary experiments. The selection of concentrations for the micronucleus assay and delayed endpoints was based on adequate cell viability following incubation.

### Micronucleus frequency

Following exposure, cells were incubated for 3 days (in experiments for immediate effects) to allow micronuclei to form during the subsequent cell divisions. The number of micronuclei and relative cell survival was assessed using flow cytometry (Bryce et al. 2007). Cells were stained as previously described (Luukkonen et al. 2011). Briefly, ethidium monoatside (EMA, 8.5 µg/mL, Invitrogen Corporation/Molecular Probes, Eugene, Oregon, USA) was used to stain nuclei from cells with a compromised cell membrane (necrotic and apoptotic cells). The second staining with SYTOX Green (0.4 µM, Invitrogen Corporation/Molecular Probes, Eugene, Oregon, USA) after lysing the cells tinted all chromatin. Therefore, micronuclei and nuclei from healthy cells were stained only with SYTOX green and were distinguishable from the nuclei and pieces of nuclei from dying cells stained with EMA. Fluorescent counting beads (Peak Flow, Green Flow cytometry reference beads, 6 µM; Invitrogen Corporation, Eugene, Oregon, USA) were included in all samples; relative cell survival was calculated from nuclei to bead ratios to monitor that no overtly toxic exposures were performed (data not shown). The samples were analyzed using a flow cytometer (Becton Dickinson FACSCalibur, San Jose, CA, USA) equipped with Cell Quest Pro (v. 5.2.1, BD Biosciences, USA) and 20,000 gated nuclei were acquired from each sample.

### Mitochondrial superoxide production

Mitochondrial O_2_^•−^ production was measured using a fluorescent probe, MitoSOX™ Red mitochondrial superoxide indicator (Molecular Probes, Invitrogen, Paisley, UK). After exposure, 1 μM MitoSOX in HBSS was added to the cells on 48-well plates and the plates were then incubated at 37 °C for 30 min. The fluorescence was measured at an excitation wavelength of 492 nm and emission wavelength of 595 nm (Tecan Infinite F200 pro, Grödig, Austria).

### Mitochondrial membrane potential

Mitochondrial membrane potential was measured using JC-1 (5,5',6,6'-tetrachloro-1,1',3,3'-tetraethylbenzimidazolylcarbocyanine iodide; Molecular Probes, Eugene, Oregon, USA). Briefly, 5 µg/mL JC-1 in DMEM was added to the wells of a 48-well plate, which was then incubated at 37 °C for 15 min. Next, cells were washed twice with warm (37 °C) PBS. The fluorescence was measured at an excitation wavelength of 540 nm and emission wavelength of 595 nm (Tecan Infinite F200 pro, Grödig, Austria).

### Mitochondrial activity

Mitochondrial activity was assayed using thiazolyl blue tetrazolium bromide (MTT, Sigma, St. Louis, USA). During the experiments 30 µL of 5 mg/mL MTT was added to 300 µL of medium 2 h prior to the end of the incubation or exposure period. After 2 h of incubation, medium and MTT were removed from the wells of a 48-well plate and 450 µL of DMSO was added into the wells. This was followed by shaking the samples on a plate shaker for 1 min and measuring the absorbance at 550 nm (Tecan Infinite F200 pro, Grödig, Austria).

### Statistical analysis

A two-way ANOVA was used for statistical analysis, with chemical concentrations as a fixed factor and replicate as a random factor. Chemical concentrations were included in the analysis to test the possible trend of the changes. Replicate was included as a random factor in the analysis because the replicates statistically significantly differed from each other in several cases. Post-hoc tests were performed using the least significant difference (LSD) test. The analysis was performed using the general linear model procedure of SPSS for Windows release 19 (SPSS Inc., Chicago, Illinois, USA) using raw or logarithm-transformed (micronucleus data) values. All experiments were replicated three times (each experiment consisted of three parallel samples, except for micronuclei experiments with two parallel samples). A *p* value of less than 0.05 was considered as a statistically significant difference.

## Results

In this study, we explored the effects of hydroxyurea and zeocin on mitochondrial integrity and genomic stability (measured by changes in micronucleus frequency). The mitochondrial integrity was assessed by the measurement of mitochondrial superoxide level, membrane potential, and mitochondrial activity. The measurements were performed immediately, 8 days, and 15 days following the exposures.

### The effects of hydroxyurea

**Fig. 2:**
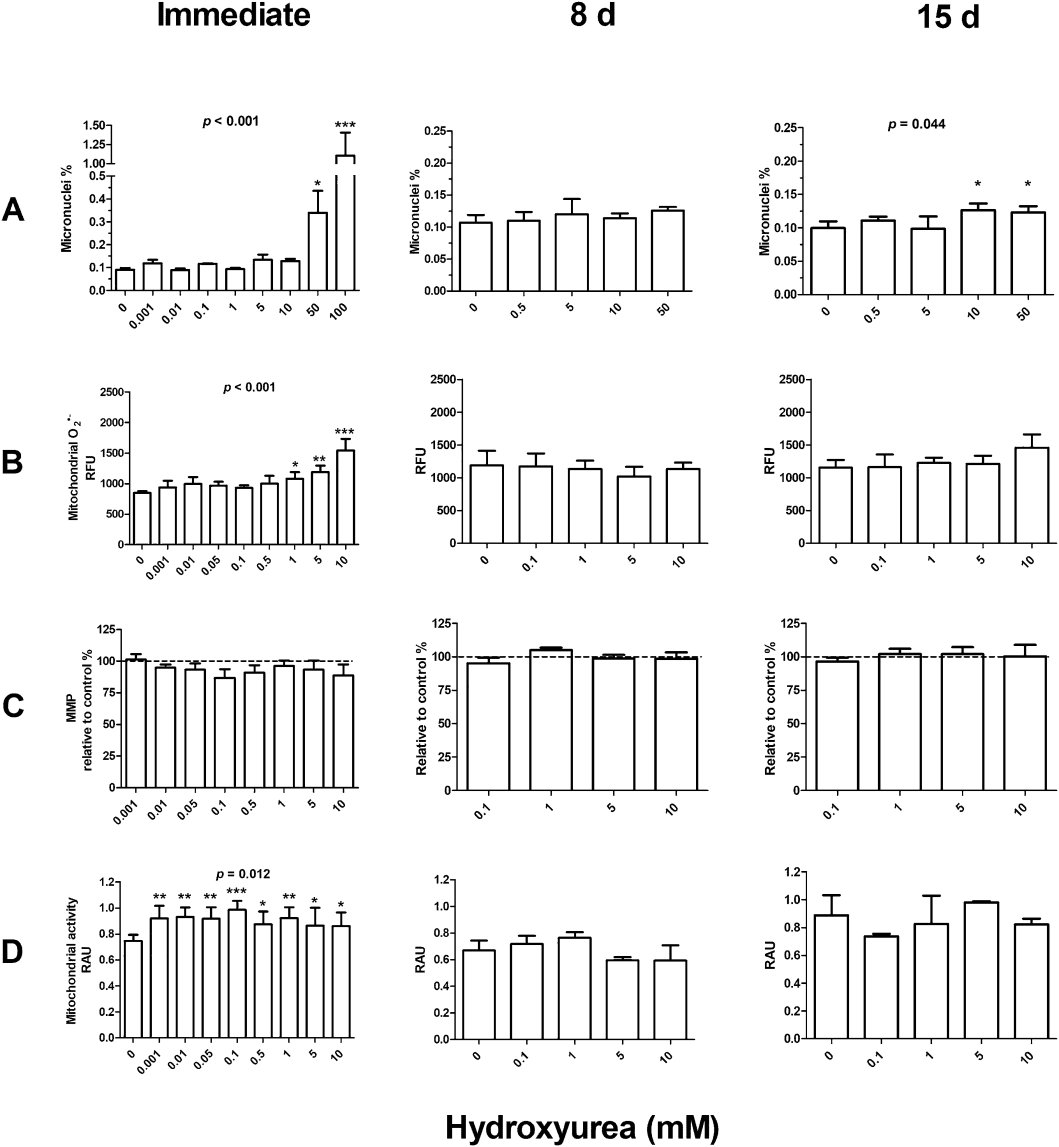
Effects of hydroxyurea on **A** micronucleus frequency, **B** mitochondrial superoxide levels, **C** mitochondrial membrane potential (MMP), and **D** mitochondrial activity immediately, 8 days, or 15 days following 3 h exposure of human SH-SY5Y neuroblastoma cells at concentrations from 0.001 to 100 mM. In the case of micronucleus frequency, immediate responses were measured at 3 days to allow formation of micronuclei, while other endpoints were measured instantly after the exposure. The *p* values provided in the figure represent the overall effect of hydroxyurea. The significances for the individual concentrations are indicated by asterisks: * = *p* < 0.05, ** = *p* < 0.01, *** = *p* < 0.001. The data are expressed as mean ± SEM, *n* = 3. RFU = Relative Fluorescence Unit, RAU = Relative Absorbance Unit

A dose-dependent increase of micronucleus frequency was observed in hydroxyurea-treated cells in the experiments reflecting immediate responses (*p* < 0.001, Fig. 2 A). Of the individual concentrations, 50 and 100 mM hydroxyurea increased the micronucleus frequency in comparison to the control group. At 8 days post exposure, hydroxyurea did not affect the micronucleus level, but an increased level was observed at 15 days post exposure (*p* = 0.044). The increase was statistically significant in both the 10 and 50 mM groups. There was no clear linear dose–response, and the effect sizes of the 10 mM and 50 mM hydroxyurea concentrations were moderate (~ 27% and ~ 23%, respectively).

Hydroxyurea increased the level of mitochondrial superoxide dose-dependently immediately after the exposures (*p* < 0.001, Fig. 2 B). This increase was statistically significant at 1, 5, and 10 mM. No significant effects of hydroxyurea on mitochondrial superoxide levels were observed at 8 days or 15 days post exposure.

The MMP level was not altered by hydroxyurea immediately after the exposure or at 8 or 15 days post exposure (Fig. 2 C).

Mitochondrial activity was elevated by hydroxyurea immediately after the exposures (*p* = 0.012, Fig. 2 D). The effect was significant at all concentrations. No effects on mitochondrial activity were observed at 8 or 15 days post exposure.

### The effects of zeocin

**Fig. 3:**
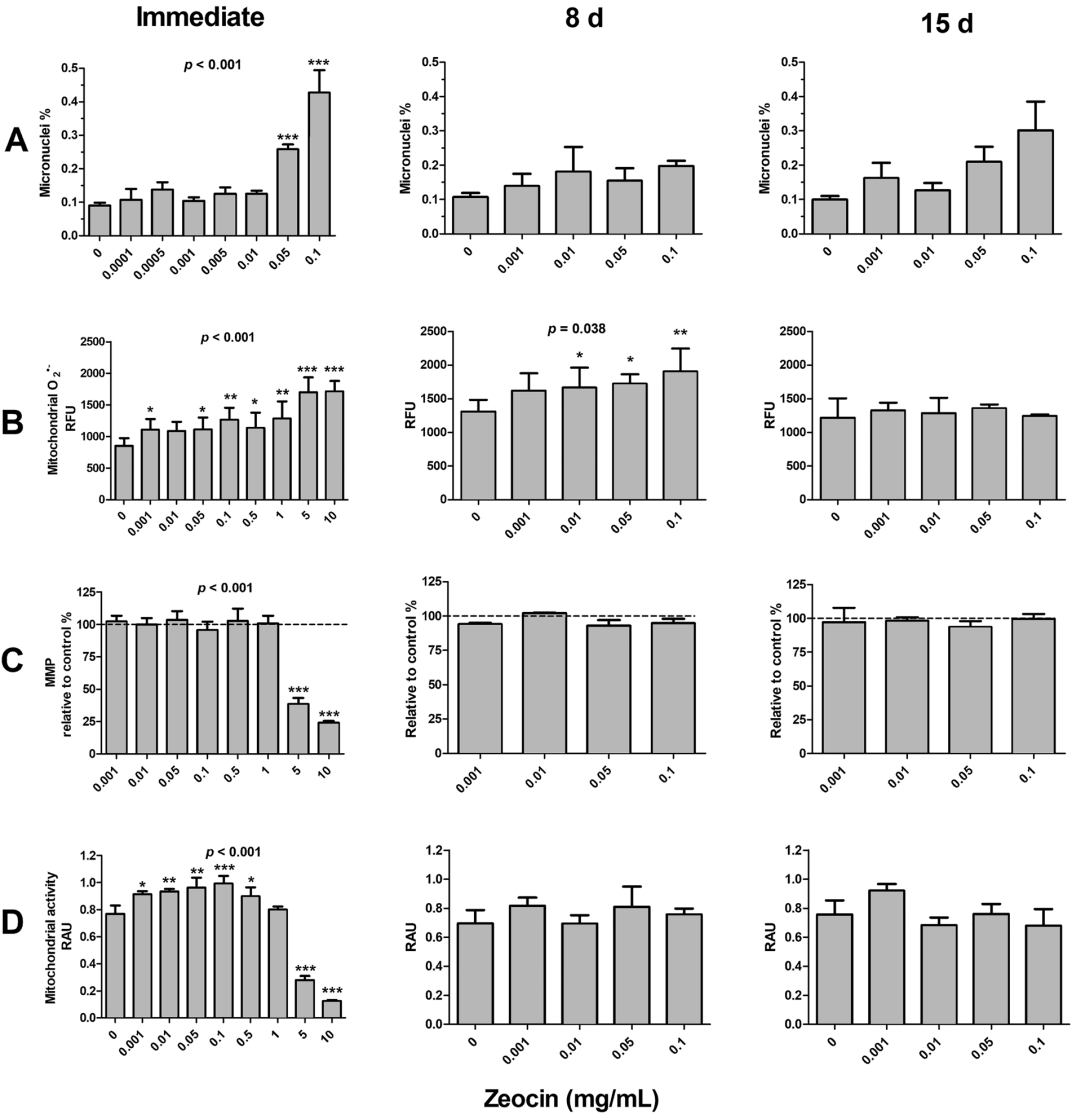
Effects of zeocin on **A** micronucleus frequency, **B** mitochondrial superoxide level, **C** mitochondrial membrane potential (MMP), and **D** mitochondrial activity immediately, 8 days, or 15 days following 3 h exposure of human SH-SY5Y neuroblastoma cells at concentrations from 0.001 to 10 mg/mL. In the case of micronucleus frequency, immediate responses were measured at 3 days to allow formation of micronuclei, while other endpoints were measured instantly after the exposure. The *p* values provided in the figure represent the overall effect of zeocin. The significances for the individual concentrations are indicated by asterisks: * = *p* < 0.05, ** = *p* < 0.01, *** = *p* < 0.001. The data are expressed as mean ± SEM, *n* = 3. RFU = Relative Fluorescence Unit, RAU = Relative Absorbance Unit

The micronucleus frequency was increased by zeocin in the experiments reflecting immediate responses (*p* < 0.001, Fig. 3 A). This increase was statistically significant at 0.05 and 0.1 mg/mL. Zeocin did not significantly increase micronucleus frequency at 8 days. At 15 days post exposure, an increasing trend with increasing zeocin concentrations was observed, the level of micronuclei being three-fold higher at 0.1 mg/mL than in the control group. However, the trend was not statistically significant (*p* = 0.101).

The level of mitochondrial superoxide increased immediately following exposure to zeocin (*p* < 0.001, Fig. 3 B). The increase was statistically significant at 0.001, 0.05, 0.1, 0.5, 1, 5, and 10 mg/mL. Increased levels of mitochondrial superoxide were observed also at 8 days after exposure to zeocin (*p* = 0.038). This increase was statistically significant at 0.01, 0.05, and 0.1 mg/mL. At 15 days, however, mitochondrial superoxide levels did not differ between in zeocin-exposed and control cells.

The level of MMP was decreased by zeocin immediately after the exposure. The overall effect and the effects of 5 and 10 mg/mL concentrations were significant (*p* < 0.001). No effects on MMP level were observed at 8 or 15 days after exposure to zeocin.

Mitochondrial activity was affected by zeocin immediately after the exposure (*p* < 0.001, Fig. 3 D). Zeocin concentrations of 0.001, 0.01, 0.05, 0.1, and 0.5 mg/mL increased mitochondrial activity, while decreased activities were observed at the concentrations of 5 and 10 mg/mL. Zeocin did not alter mitochondrial activity at 8 and 15 days post exposure.

## Discussion

In this study, we investigated whether the loss of mitochondrial integrity was involved in IGI. The study involved two chemicals that mimicked the DNA-damaging effects of ionizing radiation: one causing DNA damage via depletion of the nucleotide pool (hydroxyurea) and one causing DNA double-strand breaks (zeocin). As expected, both chemicals caused immediate genomic damage observable as increased frequency of micronuclei. The immediate responses to both hydroxyurea and zeocin included changes in mitochondrial activity, mitochondrial superoxide production. Zeocin modified also the mitochondrial membrane potential.

The delayed effects included increased level of micronuclei 15 days following exposure to hydroxyurea. Although hydroxyurea has previously been shown to cause genetic damage after continuous exposure for 4 weeks (Pruitt et al. 2017), this is the first study to our knowledge to report increased genetic damage many cell generations after a short initial exposure, consistent with the onset of IGI. This effect did not seem to follow a classical dose–response pattern: the effect sizes for the 10 mM and 50 mM hydroxyurea concentrations were similar and rather moderate (27% and 23%, respectively, Fig. 2 A). The lack of a classical rising dose–response and only moderate effect size are consistent with earlier findings on IGI resulting from exposure to radiation and chemicals (Korkalainen et al. 2012; Huumonen et al. 2014). The finding that delayed induction of micronuclei was observed only in hydroxyurea-exposed cells might suggest that depletion of the nucleotide pool can play a role in IGI. However, given the trend seen 15 days after exposure to zeocin (Fig. 3 A), the lack of a statistically significant increase of micronuclei does not allow inferring that zeocin does not induce genomic instability; we can just conclude that such an effect could not be shown in this study. Additional IGI studies using zeocin would be helpful.

The difference between the immediate effects of hydroxyurea and zeocin on mitochondria offers important insights. Both hydroxyurea and zeocin increased mitochondrial superoxide production in a dose-dependent manner immediately after exposures (Figs. 2 and 3 B). Superoxide production induced by 5 and 10 mg/mL zeocin was nearly equivalent to that induced by 10 mM hydroxyurea. However, the effects on other mitochondria-related parameters were markedly different. The 5 and 10 mg/mL zeocin treatments collapsed MMP and mitochondrial activity level (Fig. 3 C and D), but 10 mM hydroxyurea did not significantly modify the MMP level and mitochondrial activity increased (Fig. 2 C and D). It should be noted that the method (MTT) for assessing mitochondrial activity in this study is also commonly used as a measure for cellular viability, and the high-concentration zeocin observations probably indicate decreased cell viability. Overall, the results demonstrate that increased superoxide levels induced by different chemicals do not necessarily have similar manifestations in mitochondria, so this connection may not be as straightforward as commonly described.

Concerning measurements of mitochondrial integrity at the later time points, the only positive finding was an increase in superoxide production 8 days after exposure to zeocin. Confirmation of this finding will require independent testing for reproducibility. Importantly, indicators of mitochondrial dysfunction were not affected 15 days after exposure, the time when IGI became manifest. Thus, the present results do not support induction of persistent mitochondrial degeneration by short-term exposure to hydroxyurea or zeocin.

Understanding the mechanisms of persistent mitochondrial dysfunction is essential for cancer research. Several research groups (Ishii et al. 2006; Van Houten et. 2006; Ott et al. 2007) have proposed that the malfunction of the mitochondrial electron transport chain can lead to a “vicious cycle of ROS production”. According to this hypothesis, a dysfunction in the mitochondrial electron transport chain would result in elevated ROS levels, which in turn would cause additional damage to mitochondria, leading again to enhance ROS production and to mitochondrial degeneration. This “vicious cycle” may be important in cancer biology, and it has been hypothesized to be involved also in IGI (Kim et al 2006a, b). The present results do not support an essential role for mitochondrial dysfunction in IGI, as delayed induction of micronuclei (consistent with IGI) was observed in hydroxyurea-exposed cells without persistent changes in mitochondrial function. Notably, in addition to hydroxyurea, we have previously shown IGI by non-ionizing radiation in SH-SY5Y cells (Luukkonen et al 2014; Kesari et al. 2015), but the applicability of the present finding to ionizing radiation, or other exposure agents, needs to be explored in additional studies.

In conclusion, an increase in micronuclei was observed in the progeny of hydroxyurea-exposed cells, indicating manifestation of IGI. Both hydroxyurea and zeocin affected mitochondrial function immediately after exposure, but these transient responses did not lead to persistent mitochondrial dysfunction. Although we cannot exclude the involvement of immediate mitochondrial responses in the initiation of IGI, its manifestation does not require persistent degradation of mitochondrial function in human SH-SY5Y neuroblastoma cells.

## Data Availability

The original raw data will be stored at UEF’s open access repository after publication.
