# Correction to: Genomic instability induced by radiation-mimicking chemicals is not associated with persistent mitochondrial degeneration

**DOI:** 10.1007/s00411-021-00952-4

**Published:** 2021-12-02

**Authors:** Jukka Luukkonen, Anne Höytö, Miiko Sokka, Juhani Syväoja, Jukka Juutilainen, Jonne Naarala

**Affiliations:** 1 Department of Environmental and Biological Sciences, University of Eastern Finland, Yliopistonranta 1, P.O. Box 1627, 70211 Kuopio, Finland; 2 STUK-Radiation and Nuclear Safety Authority, Helsinki, Finland; 3 Department of Environmental and Biological Sciences, University of Eastern Finland, Joensuu, Finland; 4 Department of Molecular Biology, Cell Biology and Biochemistry, Brown University, Providence, RI USA; 5 Institute of Biomedicine, University of Eastern Finland, Kuopio, Finland

## Correction to: Radiation and Environmental Biophysics 10.1007/s00411-021-00927-5

Authors would like to correct the error in given name and family name which is incorrect in their publication. The correct version of author names updated.

The original article has been corrected.

